# Differential activation of nerve fibers with magnetic stimulation in humans

**DOI:** 10.1186/1471-2202-7-58

**Published:** 2006-07-24

**Authors:** Eric C Tuday, Kenneth S Olree, Kenneth W Horch

**Affiliations:** 1 Department of Bioengineering, University of Utah, Salt Lake City, Utah, USA

## Abstract

**Background:**

Earlier observations in our lab had indicated that large, time-varying magnetic fields could elicit action potentials that travel in only one direction in at least some of the myelinated axons in peripheral nerves. The objective of this study was to collect quantitative evidence for magnetically induced unidirectional action potentials in peripheral nerves of human subjects. A magnetic coil was maneuvered to a location on the upper arm where physical effects consistent with the creation of unidirectional action potentials were observed.

Electromyographic (EMG) and somatosensory evoked potential (SEP) recordings were then made from a total of 20 subjects during stimulation with the magnetic coil.

**Results:**

The relative amplitudes of the EMG and SEP signals changed oppositely when the current direction in the magnetic coil was reversed. This effect was consistent with current direction in the coil relative to the arm for all subjects.

**Conclusion:**

A differential evocation of motor and sensory fibers was demonstrated and indicates that it may be possible to induce unidirectional action potentials in myelinated peripheral nerve fibers with magnetic stimulation.

## Background

The ability to produce unidirectional action potentials (UAPs) in peripheral nerves by external stimulation would have numerous potential clinical applications. For instance, spasticity is an abnormally increased amount of muscle tone associated with an exaggerated stretch reflex. Spasticity can cause severe debilitations such as a reduced range of motion in a joint and the development of soft or hard tissue contractures. Many methods have been developed to eliminate or reduce spasticity. One of these methods involves electrically stimulating the nerve such that the resulting action potential travels in only one direction, and using them to create collision blocks in peripheral motor nerves [1-3]. However, this method is unattractive in that the electrodes must be placed close to or on the nerve, an invasive and potentially painful procedure. If UAPs could be generated via large time-varying magnetic fields, it might be possible to reduce or eliminate spasticity in a non-invasive and pain-free manner.

Magnetic stimulation is a clinically accepted means of eliciting action potentials in the human body. The basic designs of magnetic stimulators and the mechanisms of activation are well documented [4-7]. However, nobody has fully addressed the issue of magnetically induced UAP's. Indeed, it is generally thought that it cannot be done with today's commercially available magnetic stimulators because the stimulus produced is too weak and too short in duration to create and sustain an anodal block [8]. Though a mechanism has been suggested for UAP genesis in the motor cortex using magnetic stimulation [9] there have been no data presented to specifically address the idea of magnetically induced UAPs in the peripheral nervous system outside of our own findings [10]. However, there are published results that are consistent with the method of UAP genesis that we hypothesize [11,12].

One hypothetical method of UAP generation is by an anodal block of a bi-directional action potential on one side of the cathode. The anodal block works by causing the interior of the cell to become more negative relative to the extracellular space; displacing the membrane potential further from the threshold. If the membrane potential is lowered far enough away from threshold, the propagating action potential will be unable to raise the membrane potential above threshold and fail to conduct across the hyperpolarized region. The anodal region and the site of stimulation interchange positions by changing the direction of current in the coil [13,14].

Previous research has demonstrated that the amplitude of a propagating action potential in a bullfrog sciatic nerve on either side of the stimulation site is dependent on current direction in the stimulation coil, is reversed upon current reversal, and that the recording site with the smaller amplitude action potential is on the side of the virtual anode [14]. This supports the idea of UAP generation via anodal block.

Recently, it has been shown that the amplitude of the compound muscle action potential is dependent on the direction of current in the stimulating coil [12], and that the amplitude of the M-wave generated by magnetic stimulation at the elbow with a circular coil is dependent on current direction and that the site of the virtual cathode changes with current direction [11]. However, neither study measured the amount of afferent neural activity generated by magnetic stimulation and how it depended on current direction.

Previous unpublished results in our lab suggested that UAPs may be produced in the upper arm as a result of magnetic stimulation. Upon stimulation with a particular current direction in the coil, subjects exhibited forearm muscle contraction and reported little or no sensation in the hand. When the current direction was reversed in the coil (effectively reversing the current direction in the tissue), the forearm muscle contraction lessened or ceased and the subjects reported a stronger sensation. These qualitative observations indicate that magnetic stimulation might induce UAPs in the periphery, with the direction of propagation dependent on the current direction in the stimulating coil. The primary goal of the present study was to obtain quantitative measures of efferent (electromyographic potentials) and afferent (somatosenory potentials) activity during magnetic stimulation as a function of current direction in the coil.

## Results

**Figure 1:**
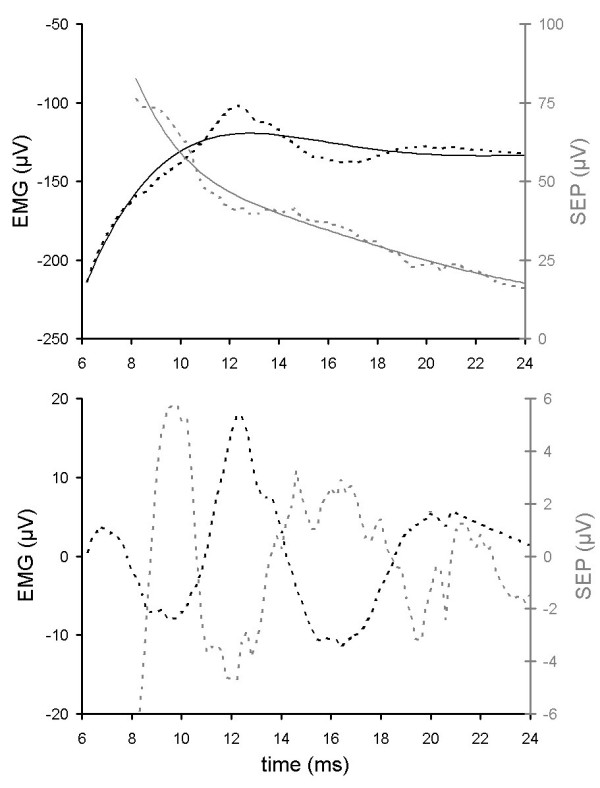
Top panel: raw data traces of an EMG signal (dotted black line, left axis) and an SEP signal (dotted gray line, right axis) from subject 39, and corresponding baseline curve fits (light lines, both 8^th ^order polynomials). Bottom panel: signals after subtraction of baseline curve fits.

**Figure 2:**
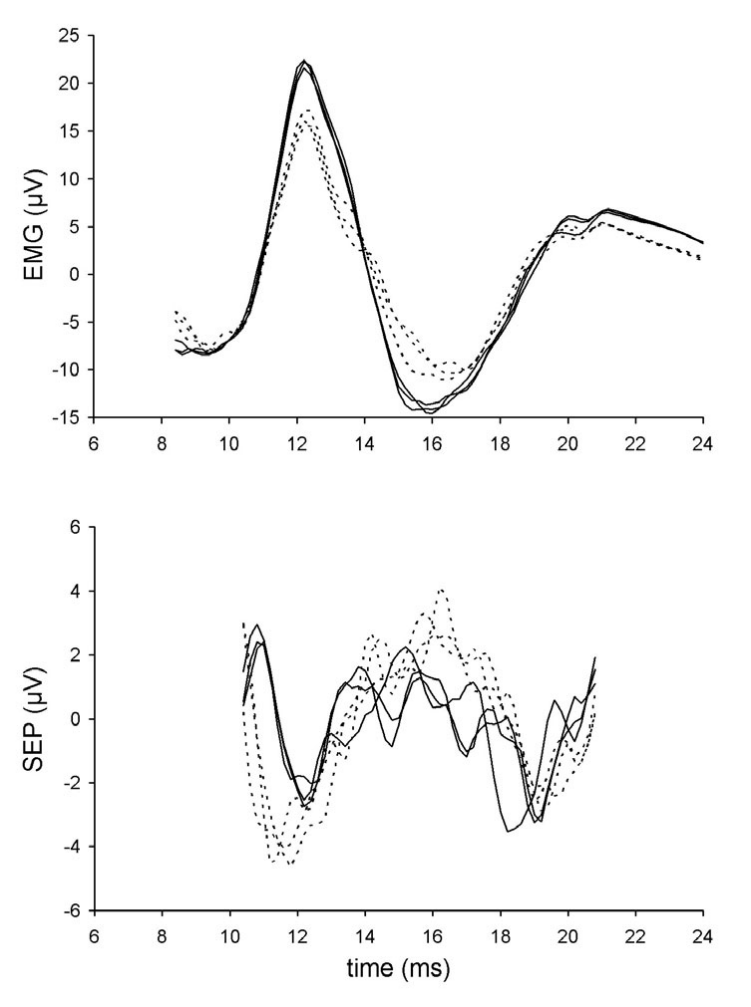
Boxcar filtered EMG (top panel) and SEP (bottom panel) data from subject 39. Solid traces are responses from CW current flow, dotted traces are from CCW current flow.

**Figure 3:**
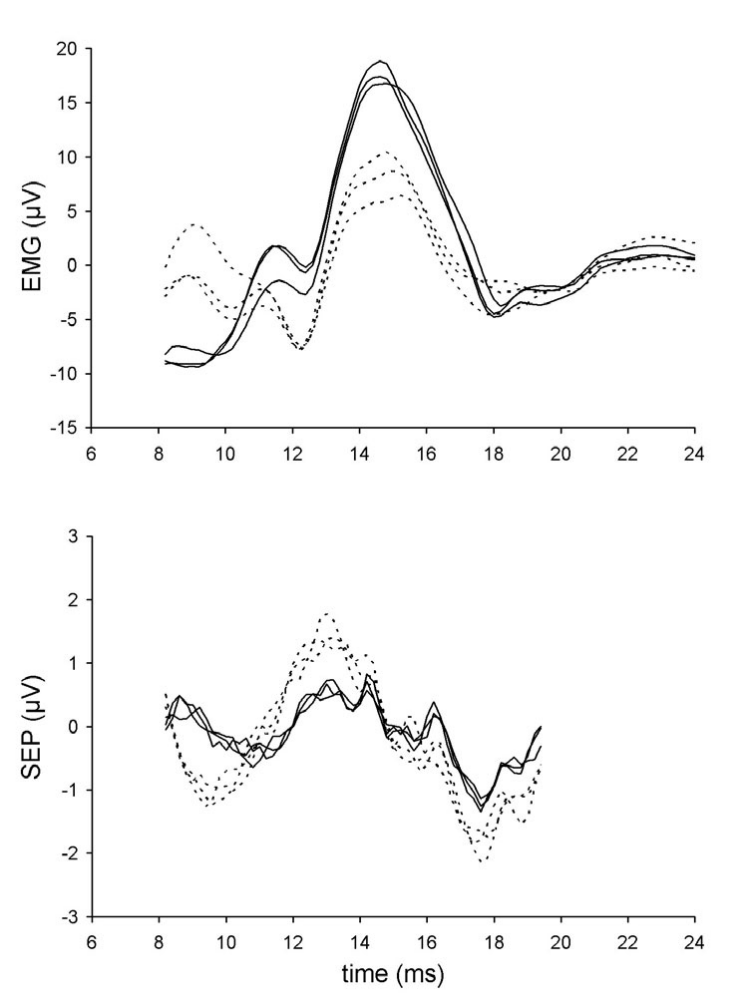
Data from subject 52. Format as in Fig. 2.

Figure 1 shows typical recordings of electomyographic potentials (EMG) recorded from the forearm and somatosensory potentials (SEP) recorded from the scalp in response to magnetic stimulation of the upper arm with a circular coil. Figure 2 shows boxcar filtered data from the same subject where the M/S ratios (normalized EMG/normalized SEP) for both current directions closely match the mean ratios of all 20 subjects. Figure 3 shows a different subject where a larger difference between the ratios was observed. Note that in both cases, CW (clockwise) current produced a larger EMG than did CCW (counter-clockwise) current in the coil, while CCW stimulating current produced a larger SEP than did CW current.

All 20 of the subjects had more EMG activity and less SEP activity when stimulating with a CW current direction compared to the CCW current direction (p < 1E-6, binomial test). This held true for each of the three coils. Put another way, M/S was always greater than 1 for CW current flow and less than 1 for CCW current flow (Table 1). The magnitude of this difference was significant for 18 of the 20 subjects (p < 0.05, t-test).

As for the effectiveness of the coils on the subjects, coil 3 (Table 2) was the most effective. In 13 of the subjects, it produced the largest contrast between the ratios. Coil 2 was responsible for producing the largest contrast between ratios in 6 of the subjects while coil 1 was best in only one subject.

The mean of the M/S ratios for all subjects was 4.5 times larger for CW stimulation than for CCW stimulation (Table 1). This value is an underestimate of the actual magnitude of the difference, since the ratio of the two values is bounded on the low end by 0, but unbounded on the upper end. If there was no effect due to current direction, or current direction affected EMG and SEP equally (e.g., one direction was more effective in eliciting neural responses than the other), the mean M/S ratio would be the same (specifically, it would have a value of 1) for both current directions. To provide a symmetrical distribution around this mean value of 1, and to allow statistical analysis of the data, the ratios were log transformed. Note that, since the data were log transformed, one needs to look at the ratio of the CW to CCW M/S ratios, not the difference between the two.

The log transformed data showed that the mean M/S ratio for CW stimulation was 5.25 that of the ratio for CCW stimulation, with a 99% confidence interval of 4.19 to 6.59 (Tukey's Honestly Significant Difference). A multi-way ANOVA test on the log transformed data from all 20 subjects gave a p < 2.2E-16.

## Discussion

The 99% confidence interval of the CW to CCW M/S ratios does not contain the value 1. Therefore we conclude that there is a difference in relative evocation of EMG and SEP activities between the two current directions.

Since the distribution of motor and sensory activity for a given subject matched the innervation pattern of a single (median or ulnar) nerve, we conclude that this effect is not likely to be due to evoking activity in a predominantly motor nerve with CW current, and a predominantly sensory nerve with CCW current. This scenario is rendered even less likely when one considers the fact that changing the current in the coil from CCW to CW always increased EMG activity and decreased SEP activity. Given the variability between subjects in the precise anatomical locations of the peripheral nerves in the arm and our procedure by which coil placement was determined without reference to which current direction had which dominant effect, it is difficult to imagine that all 20 subjects would show the same dependency on current direction.

Furthermore, we believe the possibility that current in one direction was more effective in activating nerve fibers than current in the other direction, and that differences in fiber sizes for motor and sensory axons resulted in relatively more recruitment of one population than the other in the more effective direction, is low based on the observations made when changing the current direction in the coil. If this were the case, both EMG and SEP signals would have increased with the more effective direction, rather than one going up and the other going down.

Similarly, we believe that observed distribution of M/S ratios makes unattractive the suggestion that CW versus CCW currents preferentially activate different fascicles (sensory versus motor) within a given nerve. While it is possible to selectively activate different fascicles within a peripheral nerve using extraneural stimulation through a multi-contact cuff electrode [15,16], the variability of fascicular distribution within peripheral nerves [17] means that this process has to be fine tuned on an individual subject basis. Again our placement of the coil was based simply on seeing an effect, not on the basis of which current direction was more effective for sensory or motor stimulation. Thus, if there was differential activation of motor versus sensory fascicles with different current directions, we would expect to see a random distribution of M/S ratios as a function of current direction across the subject pool (i.e., a random distribution of M/S ratios would be expected if stimulating with CW coil current produced greater motor fiber activation in some subjects and greater sensory fiber activation in other subjects as compared to stimulation with CCW current direction) However, stimulating with CW coil current resulted in greater motor activation and lesser sensory activation as compared to the CCW current direction in all subjects and the M/S ratios were comparatively high for CW and low for CCW for all subjects.

We note also that work by others has demonstrated variations in EMG amplitude with changes in coil current direction consistent with our findings [11,14], and observations of preferential motor cortex activation have been explained by an anodal block model [9].

Though we find the results consistent with a model of UAP generation via anodal block, we have no direct evidence that an area of hyperpolarization was generated so as to produce an anodal block in the nerves stimulated, and our results should be considered with that in mind.

## Conclusion

It is apparent that there was a differential activation of motor and sensory nerves upon stimulation with a magnetic coil. Based on the results obtained in these experiments, and the results from *in vitro *experiments in our lab [10], we find that it is possible that the data presented reflects the creation of UAPs via an anodal block mechanism. However, since we provide no direct evidence to support this, it is possible that the observed effect may be due to one of the other possible explanations discussed earlier.

Still, all of the observations made during these experiments are consistent with our model of UAP generation. For one, since the change in EMG and SEP amplitudes varied consistently with the direction of the current in the coil relative to the arm, the blocking mechanism must be dependent on current direction, as is the position of the anodal region relative to the cathodal region in the stimulated nerve [13]. Additionally, we have developed a detailed computer model to analyze field distributions with circular coils applied to the upper arm in humans. This model shows that under the proper conditions, unidirectional action potentials can be produced by such coils, a finding that has been confirmed with excised nerves placed in a geometrically well-defined environment [10]. However, there are variations in conductor inhomogeneities on an individual basis that are not accounted for in our computer model, which can greatly influence current flow and the site of excitation [18].

## Methods

**Figure 4:**
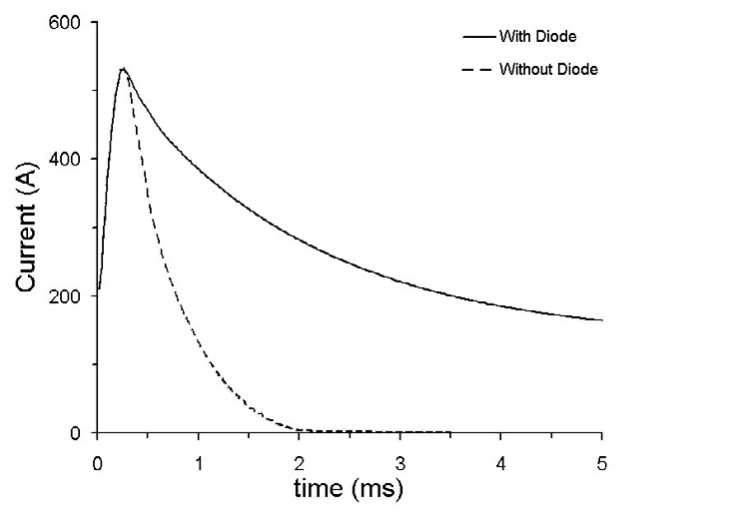
Coil current profiles with and without the diode placed across the coil (data taken with coil 3 described in Table 2).

Three stimulating coils were used for the study. All three were circular in design with varying layers and turns (Table 2). The stimulator was custom designed by Maxwell Labs (San Diego, CA) and passed a maximum charge of 0.3 coulombs through an IGBT switch. A diode was placed in parallel with the coil [10], which favorably changed the current profile in the coil by reducing the rate at which the coil current decayed (Fig. 4). The induced current density in the tissue is proportional to the time rate of change of current in the coil; so when di/dt in the coil is negative, the effects elicited in the nerve by the initial rise of current in the coil are negated. Reducing the negative di/dt decreases the negation of the effects.

This study was approved by the University of Utah's Institutional Review Board and all 20 subjects in this study gave informed consent. Each subject was randomly assigned an ID number and seated comfortably in a chair. The subject was unaware of the goals of the study to prevent any bias.

Electromyographic (EMG) recordings were made by placing two recording electrodes (Kendall Biotac Ultra 7563) on the skin surface directly over the proximal and distal portions of the bellies of the forearm flexor muscles, and a ground electrode was placed on the elbow. Before the electrodes were placed, the skin was thoroughly cleaned using an alcohol swab. The electrodes were connected to a WPI DAM-6 differential amplifier with a voltage gain of 100 and bandpass filtering set at 10–3,000 Hz [19].

Somatosensory evoked potential (SEP) recording electrodes (copper metal disks) were placed at points CP3 and CP4 as described by the international 10–20 system [20]. Conducting jelly was placed on the recording electrodes to minimize contact impedance. The electrodes were held in place by wrapping an ace bandage around the head. A ground electrode (Kendall Biotac Ultra 7563) was placed behind the left ear. The electrodes were connected to a WPI DAM-6 differential amplifier with a voltage gain of 100 and bandpass filtering set at 10–3,000 Hz [21].

Before stimulation, a thermal insulating sleeve made out of one-inch thick Styrofoam surrounded by nylon was placed on the upper, right arm of each subject as protection from the heat generated by the coil. A magnetic stimulus was presented every 0.3 s and each subject was allowed to move the coil to various positions on the arm. The subject reported on the sensation felt and forearm muscle activity was observed by the researcher. The coil was moved until a site was found where the subject reported little or no sensation and the researcher observed a strong forearm muscle contraction or, conversely, the subject reported a strong sensation and the researcher observed little or no forearm muscle contraction. The current in the coil was then reversed to see if the opposite effect was observed, i.e., if strong forearm muscle contraction would decrease and the reported sensation would increase (or vice versa). This deliberate search for a site of asymmetrical activity was necessary because UAP genesis is highly dependent on the location of the anodal block relative to the site of activation. Therefore the coil had to be carefully oriented for each subject. Care was taken to insure that the elicited motor activity and the reported sensations in a given subject corresponded to the innervation patterns of the same nerve (ulnar or median, depending on the subject).

**Figure 5:**
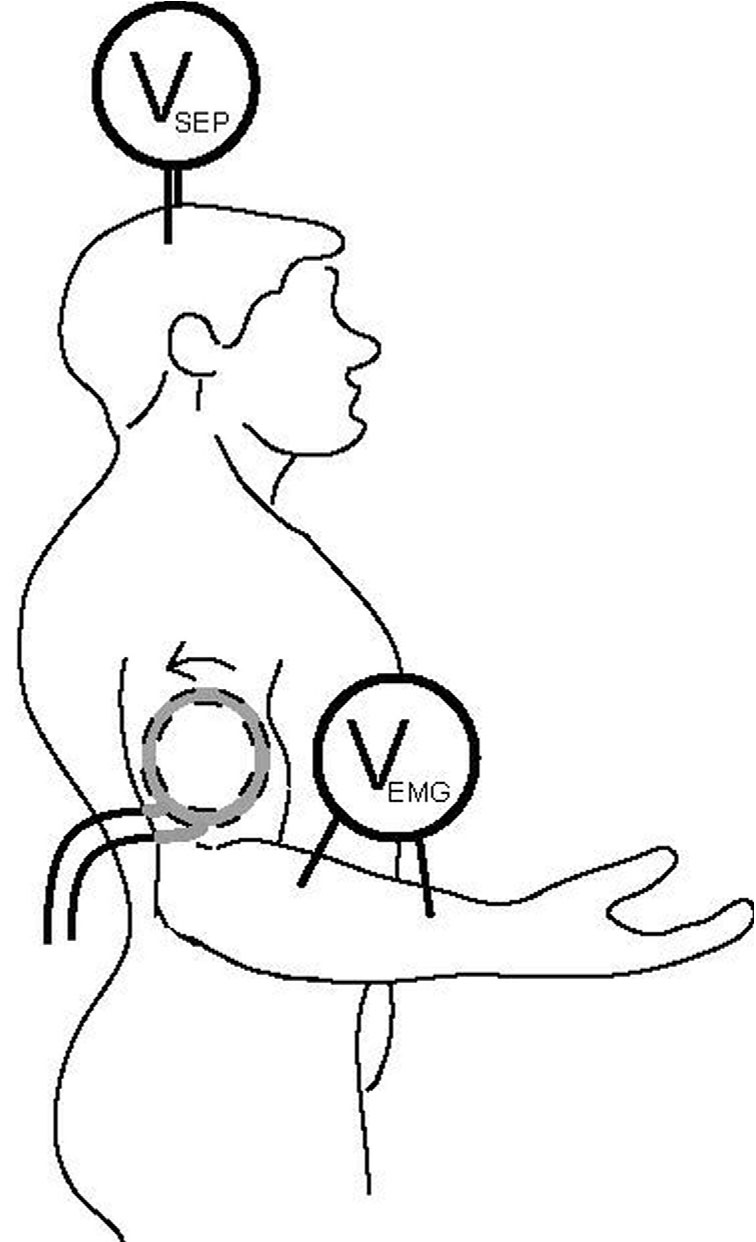
Sketch of electrode and coil placement. The coil was placed on the medial side of the right arm. The arrow indicates CW current flow in the coil.

Once a site of suspected UAP production was found, the coil was fixed in place either with a non-magnetic mechanical arm or with an Ace bandage. The favorable site for the coil was on the medial side of the arm flat against the skin for all subjects (Fig. 5); however, the exact location within this area of the arm varied between subjects. With the coil fixed in place, the subject was stimulated for a total of 6 different trials, 3 with a clockwise (CW, as viewed from the body midline) current direction in the stimulating coil and 3 trials with counterclockwise (CCW) current (Fig. 5). The order of the trials was randomly generated by a computer. All three coils were used for each subject, each being positioned independently.

The amplified EMG and SEP signals were passed to an oscilloscope that allowed visual confirmation that the signals were present, and provided further amplification (voltage gain of 100) to match the input range of the A/D card (National Instruments CB-50LP) in the computer. The signals were time aligned by using a trigger pulse generated by the magnetic stimulator, digitized at a rate of 5,000 samples/s for 50 ms, signal averaged, and stored on the computer's hard disk drive by a virtual instrument created using Labview. A total of 128 repetitions were used for each average. The entirety of the 128 repetitions constitutes one trial.

Stimulation with the magnetic coil caused a large artifact in the EMG and SEP recordings that began to diminish to baseline at about 6 ms in a steady manner. Artifact removal was accomplished by fitting the recording with a polynomial (usually a 7^th ^or 8^th ^order, depending on which fit best) and subtracting the results of that polynomial from the EMG or SEP trace (Fig. 1). Subtle variations (caused by positional differences of the coil relative to the electrodes) in the signal artifact sometimes made a 7th order polynomial a more appropriate fit. The fit was made for the entire trace (6 ms to 50 ms) to ensure that the polynomial fit was to the artifact and not to the evoked neural activity. In addition to removing the artifact, the subtraction eliminated any DC offset present in the signal. Finally, the data were smoothed with a three-sample boxcar filter.

With the artifact removed, the data within a window of 21 ms were quantified for the EMG trace and the data within a window of 16 ms were quantified for the SEP trace to give a single value for each response that represents the amount of activity present in each window. Quantification consisted of calculating a mean-squares value (proportional to signal power) for motor (M) and sensory (S) stimulation as follows:

M=∑n=1N(VEMG)n2N     Eqn. 1
 MathType@MTEF@5@5@+=feaafiart1ev1aaatCvAUfKttLearuWrP9MDH5MBPbIqV92AaeXatLxBI9gBaebbnrfifHhDYfgasaacH8akY=wiFfYdH8Gipec8Eeeu0xXdbba9frFj0=OqFfea0dXdd9vqai=hGuQ8kuc9pgc9s8qqaq=dirpe0xb9q8qiLsFr0=vr0=vr0dc8meaabaqaciaacaGaaeqabaqabeGadaaakeaacqqGnbqtcqGH9aqpdaWcaaqaamaaqahabaWaaeWaceaacqqGwbGvdaWgaaWcbaGaeeyrauKaeeyta0Kaee4raCeabeaaaOGaayjkaiaawMcaamaaDaaaleaacqqGUbGBaeaacqaIYaGmaaaabaGaeeOBa4Maeyypa0JaeGymaedabaGaeeOta4eaniabggHiLdaakeaacqqGobGtaaGaaCzcaiaaxMaacqqGfbqrcqqGXbqCcqqGUbGBcqqGUaGlcqqGGaaicqqGXaqmaaa@4734@

S=∑n=1N(VSEP)n2N     Eqn. 2
 MathType@MTEF@5@5@+=feaafiart1ev1aaatCvAUfKttLearuWrP9MDH5MBPbIqV92AaeXatLxBI9gBaebbnrfifHhDYfgasaacH8akY=wiFfYdH8Gipec8Eeeu0xXdbba9frFj0=OqFfea0dXdd9vqai=hGuQ8kuc9pgc9s8qqaq=dirpe0xb9q8qiLsFr0=vr0=vr0dc8meaabaqaciaacaGaaeqabaqabeGadaaakeaacqqGtbWucqGH9aqpdaWcaaqaamaaqahabaWaaeWaceaacqqGwbGvdaWgaaWcbaGaee4uamLaeeyrauKaeeiuaafabeaaaOGaayjkaiaawMcaamaaDaaaleaacqqGUbGBaeaacqaIYaGmaaaabaGaeeOBa4Maeyypa0JaeGymaedabaGaeeOta4eaniabggHiLdaakeaacqqGobGtaaGaaCzcaiaaxMaacqqGfbqrcqqGXbqCcqqGUbGBcqqGUaGlcqqGGaaicqaIYaGmaaa@4767@

where N is the number of samples in the time window.

The starting points of the windows were adjusted appropriately for each individual to correspond with the end of the large transient associated with the artifact, though starting points of 6 ms for the EMG trace and 8 ms for the SEP trace were typical. Data within a window of equal duration at the end of the trace were quantified in the same manner to provide a measure of noise. The noise mean-squares value was subtracted from the response value to obtain a final mean-squares value.

The M value for a single trial was normalized by dividing it by the average M value of all six trials. The S values were normalized similarly. The ratio of the normalized M value to the normalized S value was calculated for each trial. The ratios of the entire study population were log transformed and a multiway ANOVA test was conducted on the entire data set to test for statistical significance between current directions (statistics were performed using the R statistics package, a language and environment for statistical computing and graphics readily available on the World Wide Web [22]). Tukey's Honestly Significance Difference post-test was performed to obtain the 99% confidence interval of the differences in the log ratios.

## Authors' contributions

ET contributed to the experimental design, carried out all the experiments, built the data acquisition system, performed data analysis, and contributed to the manuscript. KO assisted in the experiments, data analysis, and contributed to the manuscript. KH conceived, designed, and assisted in the study and data analysis, and contributed to the manuscript. All authors read and approved the final manuscript.

## Figures and Tables

**Table 1:** Data summary. Shown are mean ± standard deviation.

**Measure**	**CW**	**CCW**
M	1.37 ± 0.26	0.62 ± 0.21
S	.699 ± 0.25	1.30 ± 0.32
M/S	2.16 ± 0.94	0.48 ± 0.19

**Table 2:** Physical characteristics of the coils. The resistance of the coils was too low (order of milliohms) to measure accurately as measurements were dominated by contact resistance.

**Coil**	**Turns per layer**	**Layers**	**Inner diameter (cm)**	**Outer diameter (cm)**	**Wire gauge**	**Inductance (μH)**
1	3	3	5.2	7.6	8	7.5
2	2	5	5.1	9.8	6	8.5
3	5	5	5.1	10.2	8	41
